# A qualitative study of foundation year two (F2) doctor's attitudes towards psychiatry carried out in Northern Ireland

**DOI:** 10.1192/bjo.2021.111

**Published:** 2021-06-18

**Authors:** Michael Doris, Kathyrn Mitchell, Damien Hughes, Lorraine Parks, Angela Carragher

**Affiliations:** 1 Belfast HSC Trust; 2 South Eastern Health and Social Care Trust

## Abstract

**Aims:**

Recruitment into psychiatry is a major issue nationally. Northern Ireland (NI) consistently punching above it's weight with psychiatry recruitment - in a region that only attracts 31.8% of F2s to enter into any training programme, Core psychiatry has been consistently oversubscribed. Here we look to examine the experiences of F2s in NI, including those who have had a placement in psychiatry and those who have not - what can we learn from NI?

**Background:**

The exposure to psychiatry during the F2 year is a crucial time for recruitment to psychiatry. In NI, where there has been an 100% fill rate at core training level for many years, trainees and consultants have pointed towards a positive experience in the F2 year.

**Method:**

Questionnaires were given out at a sample of F2 Generic Skills sessions, gathering a range of quantitative and qualitative data. A representative sample of over half of current F2s wrote about there preconceptions and experiences of psychiatry, whether they had worked in it or not. An a priori approach was taken towards generating codes as part of a framework analysis from which 4 major themes were identified.

**Result:**

93/148 F2 doctors who were approached responded to the survey of which 36.6% had experienced a Foundation placement in psychiatry. Major qualitative themes that emerged were exposure to psychiatry, the nature of working in psychiatry, being valued and stigma. Doctors who had an F2 placement were much more likely to be willing to pursue a career in it, regardless of whether they had been allocated a placement with psychiatry by choice or not.

**Conclusion:**

This survey adds to the literature that exposure to psychiatry in undergraduate and postgraduate level has a huge role in shaping attitudes towards the specialty of psychiatry, and indeed the likelihood of a foundation doctor going on to become a psychiatry trainee. Stigma in the medical profession towards mental illness and psychiatry remains prevalent.

